# The Immunopathogenesis of Neuroinvasive Lesions of SARS-CoV-2 Infection in COVID-19 Patients

**DOI:** 10.3389/fneur.2021.697079

**Published:** 2021-07-30

**Authors:** Shamila D. Alipoor, Esmaeil Mortaz, Mohammad Varahram, Johan Garssen, Ian M. Adcock

**Affiliations:** ^1^Molecular Medicine Department, Institute of Medical Biotechnology, National Institute of Genetic Engineering and Biotechnology, Tehran, Iran; ^2^Department of Immunology, School of Medicine, Shahid Beheshti University of Medical Sciences, Tehran, Iran; ^3^Clinical Tuberculosis and Epidemiology Research Center, National Research Institute of Tuberculosis and Lung Diseases, Shahid Beheshti University of Medical Sciences, Tehran, Iran; ^4^Mycobacteriology Research Center, National Research Institute of Tuberculosis and Lung Diseases, Shahid Beheshti University of Medical Sciences, Tehran, Iran; ^5^Division of Pharmacology, Faculty of Science, Utrecht Institute for Pharmaceutical Sciences, Utrecht University, Utrecht, Netherlands; ^6^Danone Nutricia Research, Utrecht, Netherlands; ^7^National Heart and Lung Institute, Imperial College London and the National Institute for Health Research Imperial Biomedical Research Centre, London, United Kingdom; ^8^Priority Research Centre for Asthma and Respiratory Disease, Hunter Medical Research Institute, University of Newcastle, Newcastle, NSW, Australia

**Keywords:** COVID-19, SARS-CoV-2-, neuroinvasive lesions, cytokine storm, IL-6

## Abstract

The new coronavirus disease COVID-19 was identified in December 2019. It subsequently spread across the world with over 125 M reported cases and 2.75 M deaths in 190 countries. COVID-19 causes severe respiratory distress; however, recent studies have reported neurological consequences of infection by the COVID-19 virus SARS-CoV-2 even in subjects with mild infection and no initial neurological effects. It is likely that the virus uses the olfactory nerve to reach the CNS and that this transport mechanism enables virus access to areas of the brain stem that regulates respiratory rhythm and may even trigger cell death by alteration of these neuronal nuclei. In addition, the long-term neuronal effects of COVID-19 suggest a role for SARS-CoV-2 in the development or progression of neurodegerative disease as a result of inflammation and/or hypercoagulation. In this review recent findings on the mechanism(s) by which SARS-CoV-2 accesses the CNS and induces neurological dysregulation are summarized.

## Introduction

The central nerve system (CNS) is protected from pathogens by effective immune responses and physical barriers including the blood brain barrier (BBB) and blood cerebral-spinal fluid (CSF) barriers (1). However, viral infection of the CNS does occur and may cause disease via direct viral-induced neuronal damage or by inflammatory- or immune-mediated pathologic mechanisms (1).

Brain cells express immune receptors that recruit leukocytes to the CNS upon detection an infection. The initial inflammatory response upon neurological infection frequently leads to an increase in the permeability of the BBB and potentially as a consequence severe neurological damage (1, 2). Viral infections can induce serious CNS injury including encephalitis or severe acute demyelinating lesions (3). In addition, viruses induce the production of chemokines and cytokines such as type I interferon (IFN) and the expression of major histocompatibility complex (MHC)-1 by neurons *in vivo* and *in vitro* (4). To control the immune response and avoid immune mediated tissue damage, the expression of anti-inflammatory cytokines including IL-10 is enhanced within the CNS upon viral infection. This tight regulation of immune responses within the CNS is important since neurons are highly specialized cells with a limited regenerative capacity (3).

Previous studies have shown the neurotropism of severe acute respiratory syndrome coronaviruses (SARS-CoVs) and highlighted their ability to cause brain infection in patients with SARS and Middle East Respiratory Syndrome (MERS) (3). Considering the high degree of similarity between SARS-CoV and SARS-CoV-2, SARS-CoV-2 should possess a similar mechanism for the induction of CNS injury and brain damage. In this review, an overview on the mechanism(s) by which SARS-CoV-2 accesses the CNS and induces neurological dysregulation is described.

### Possible Mechanisms for Entering the CNS

Neuro-invasion is a common feature of human coronaviruses (5). Autopsy findings have confirmed the presence of SARS-CoV in the brain tissue and CSF of patients with severe SARS or MERS by electron microscopy, reverse transcription PCR and by immunohistochemistry (IHC) (6–8). Interestingly, the RNA of human coronavirus OC43 (HCoV-OC43) and human coronavirus 229E (HCoV-229E) was detected in the brain tissue of multiple sclerosis (MS) patients (9, 10) and their putative role in chronic neurological disorders such as Parkinson's disease (PD), headache and MS has recently been suggested (11).

Coronaviruses are able to locate to the brain via synapse-connected routes. For example, coronaviruses may first infect peripheral nerve terminals and reach the CNS via retrograde transport (12). Neurotropism of coronaviruses was first described with swine hemaglutinating encephalomyelitis virus (HEV) (13). Oronasal inoculation of HEV in suckling piglets indicated primary viral replication in nasal epithelial cells, tonsils and the small intestine. Subsequently, the virus was transported to the CNS and brain stem via retrograde transport along the peripheral nervous system. Viral spread into the cerebrum, spinal cord and neural plexuses of the stomach was also observed in the later stages of HEV infection which affected the peristaltic function of the digestive tract, resulting in anorexia and vomiting (13).

In experimental models, HCoV-OC43 was able to reach the CNS via the olfactory route by neuron-to-neuron transmission and to replicate in the brain stem and spinal cord (14). The infectivity of adults with a normal immune system to HCoV-OC43 results in a mild chronic or latent disease but in immunocompromised individuals and children this virus may lead to lethal acute encephalitis (15). Furthermore, intranasal infection of mice with SARS or MERS-CoVs evoked rapid histopathological changes in the brain stem and thalamus (7). This further supports the concept that viral transmission to these brain areas was predominantly via the olfactory nodes (8).

Considering the high similarity between SARS-CoV and SARS-CoV2, it is expected that SARS-CoV-2 might show a similar mechanism of CNS invasion (16). Non-neural cells in the olfactory system express high levels of angiotensin-converting enzyme 2 (ACE2), the receptor for SARS-CoV-2, and for this reason can be infected by SARS-CoV-2 (17). These cells have a supportive role for mature olfactory sensory neurons (OSNs) and can transfer the virus to OSNs through axonal transport (14). This process enables the penetration of neuroinvasive viruses other than SARS-CoV-2 into the brain and the generation of a local inflammatory response which may act as trigger of neurodegenerative diseases (18, 19). Thus, the early occurrence of loss of smell or hyposmia during COVID-19 infections should be taken into consideration as a marker for subsequent CNS involvement (20).

Neuronal entry of SARS-CoV-2 may also occur via a neuropilin-1 (NRP1)-related pathway. NRP1 is a transmembrane glycoprotein receptor and can bind furin-cleaved substrates (21). NRP1 is highly expressed in endothelial and epithelial cells of the respiratory and olfactory systems (22) and in vagal and other sensory neurons (23). The coronavirus spike (S1) protein contains a furin cleavage site (24) which suggests that it has the potential to activate NRP1 and gain cell entry (25). Interestingly, monoclonal antibody against NRP1 significantly reduces viral infectivity significantly (21).

Another suggested mechanism accounting for viral entry into the brain is the systemic circulation and disruption of the BBB (26). ACE2 receptors are widespread throughout the brain stem including regions with high vascularization and a leaky BBB would allow viral infection that may subsequently induce or accelerate neurodegeneration (26). Noroviruses can infect the endothelial cells that are one of the three cell types that contribute to the BBB and increase the expression of matrix metalloproteinases (MMPs). This, in turn, decreases the expression of tight junction proteins resulting in increased permeability of the BBB (27). Furthermore, replication and budding of the virus, along with the subsequent expression of inflammation cytokines, will disrupt the normal function of the BBB and enable virus access to the CNS (15). This mechanism is observed with viruses such as hepatitis E virus (28) and encephalitic alpha viruses (29). Because of the high expression of ACE2 in cellular components of the BBB, it is suggested that the disruption of BBB by SARS-CoV-2 may play the main role in the central and peripheral nervous system damage seen in severe COVID-19 (30).

**Figure 1:**
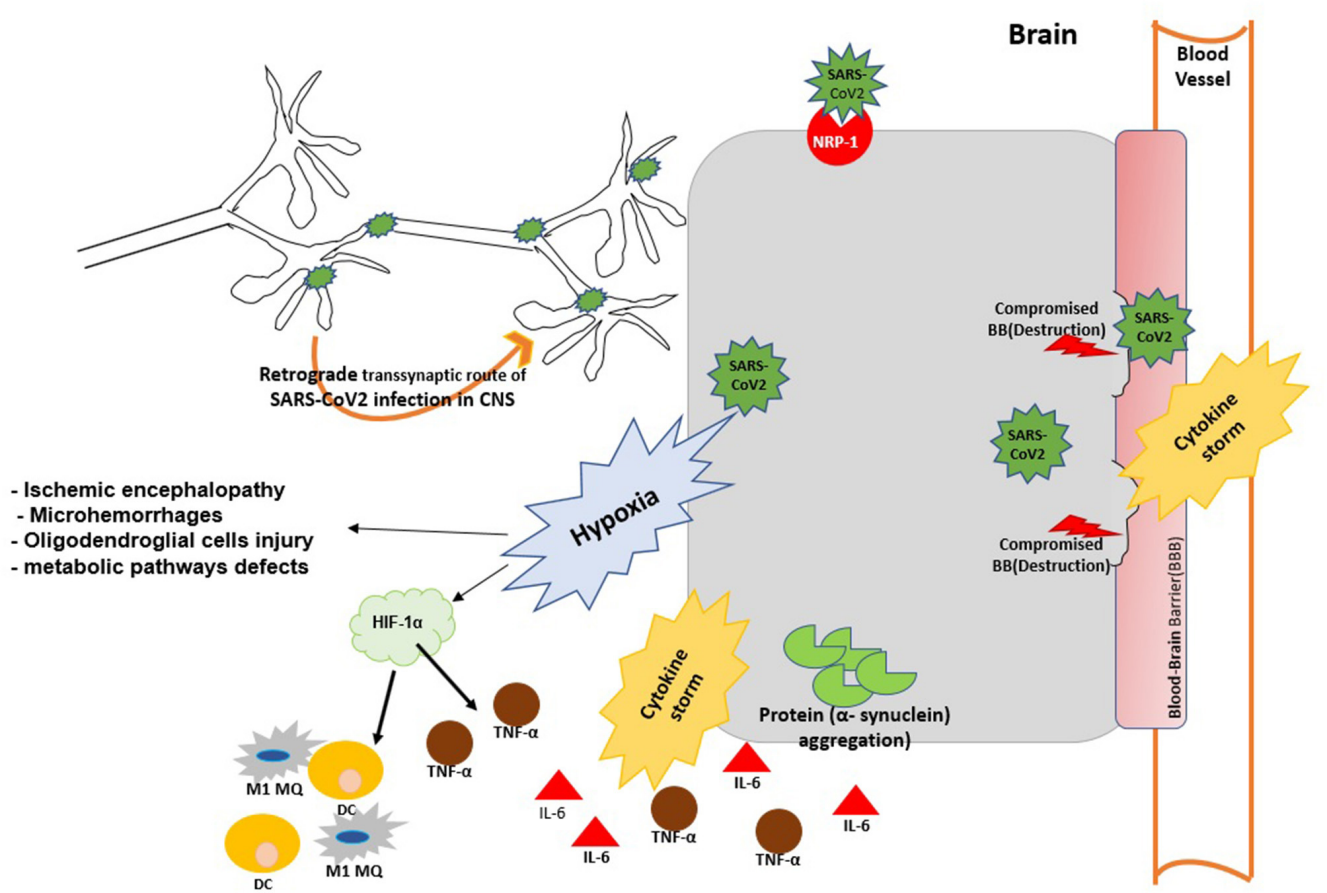
The possible mechanisms of virus entry to CNS and brain injury during SARS-Cov2 infection. SARS-Cov2 can reach the central nervous system (CNS) by retrograde transmission along the peripheral nervous system; by activation of the neuropilin-1 (NRP1) receptor; via the systemic circulation and disruption of the BBB. Viral infection can cause aggregation α-synuclein and trigger α-synucleinopthies. Viral replication triggers cytokine storm and case to vascular leakage and BBB disruption. Virus infection may trigger brain hypoxia during which hypoxia inducible factor 1 (HIF-1α) is activated in the brain and promotes the production of IL-6 TNF-α and M1 MQ polarization at the site of infection. Hypxia in the brain impairs metabolic pathways and further promotes neurodegeneration and brain injury.

Rather than destruction of the BBB, other viruses such as human immunodeficiency virus (31) and Zika virus (32, 33) enter the CNS through a Trojan horse mechanism in which they infected monocytes which shuttle them across the BBB. Coronaviruses have the same potential as HIV to constitute a reservoir in leukocytes and use them as carrier to spread into the other tissues (34). There are reports indicating that ACE2-expressing CD68^+^CD169^+^ macrophages may act as a Trojan horse and contribute to viral spread of SARS-CoV-2 (34) although this needs to be confirmed (Figure 1). Furthermore, the S1 spike protein of SARS-CoV-2 can cross the BBB and reach CNS via absorptive mediated transcytosis (AMT) in mice (35).

### Neurological Manifestations and Post-COVID-19 Neurological Syndrome

Headache, dizziness, chronic fatigue, musculoskeletal pain with fever and taste and smell disorders are reported as the most common neurological manifestations in COVID-19 patients. Dry cough is also a common symptom of COVID-19 and is considered as a neurological consequence. Viral infection of the sensory nerves and of the airway vagus nerve promote neuroinflammation and neuroimmune interactions that probably initiate cough (36).

The occurrence of cough together with fatigue, dyspnoea and pain further confirms the CNS injury in COVID-19 patients (36). Chronic fatigue and fibromyalgia (fatigue accompanied by musculoskeletal pain, sleep, memory and mood concerns) in COVID-19 patients have also been associated with alterations in the processing of pain and sensory signals by the brain (37) in these patients.

The persistence of these neurological symptoms for many months in COVID-19 recovered subjects is described as “post-COVID syndrome (PCS)” or long COVID (38, 39). Some patients also struggle with clouding of consciousness so-called mental fog (involving memory and focusing problems and a lack of mental clarity) for a long time after recovery which exert a deleterious effect on their daily activities (40, 41). The mechanism behind the post-COVID syndrome has not been completely clear but it may be the result of neuroinflammatory reactions in the brain.

Brain MRI imaging (42) in the patients with long COVID have shown cortical signal abnormalities and neuroinflammation. On the other hand PET imaging has shown bilateral hypometabolism in the olfactory gyrus, brain stem and the cerebellum (43) in patients. Analysis of 19 mild and 32 severe COVID-19 patients with no specific neurological symptoms during acute infection and no obvious lesions seen using conventional magnetic resonance imaging (MRI) 3 months after discharge indicated decreased cerebral blood flow and the presence of brain microstructure changes. Changes including decreased cortical thickness, white manner microstructure, particularly in the frontal and limbic systems, were more evident in subjects with severe disease and correlated with the degree of systemic inflammation observed (44).

ACE2 expression in the human dorsal root ganglion (DRG) sensory neurons and in P2RX3 (purinergic receptor P2X3) pain receptors and the subsequent damage of these nerves upon viral infection might be responsible for the post-COVID neurological symptoms (45, 46).

Although it is too early to describe the challenge and clinical features of PCNS as a novel syndrome there is a clear need for neurological monitoring of COVID-19 survivors and further studies to managing the future challenge in this issue.

### CNS Injury and the Immune Response in COVID-19

There is a large body of evidence suggesting a role for specific pathogens, especially viruses, in the initiation or facilitation of neurodegenerative disease. The early evidence for a viral cause of Parkinsonism was during an outbreak of encephalitis lethargica (EL) in 1918–30 that occurred during and after the 1918 influenza outbreak (47). In this case, a large number of patients with an acute episode of EL developed post-encephalitic Parkinsonism (48).

In addition, nasal administration of the highly pathogenic H5N1 influenza virus into mice results in microglial activation and α-synuclein aggregation in virus-infected brain areas that persisted long after the resolution of the infection. Infection also resulted in a significant loss of dopaminergic neurons within the substantia nigra (SN) (49).

To date, 36.4% of patients with COVID-19 have shown neurological symptoms (50) and viral particles have been detected in the postmortem brains of COVID-19 victims (26). The first case of SARS-CoV-2-associated neurological injury was a 24-year-old man with meningitis who was admitted to hospital on the 9th day after the onset of symptoms with convulsions and unconsciousness. Interestingly, SARS-CoV-2 RNA was detected in the patient's CSF but was not detectable in the nasopharyngeal swab (51).

COVID-19/SARS-CoV-2-associated neurological defects vary from non-specific to specific symptoms (52, 53) and autopsy results have shown edema and neuronal degeneration in the brain tissue of COVID-19 victims (50). However, the pathophysiological mechanism behind the SARS-CoV-2-associated encephalitis is not fully understood. The virus may induce nerve damage through several mechanisms including direct infection (54) or an immunologic response to SARS-CoV-2 infection (55).

The induction of the type I IFNs provide the first line of immune defense against CNS viral infection (56) which consequently trigger and regulate the specific adaptive immune responses to limit viral spread (57). However, other defense mechanisms may exist as well. Viral infection, for example, can lead to an aggregation of proteins including α-synuclein as an anti-viral defense mechanism, which can trigger α-synucleinopthies (58). Dopaminergic neurons are more vulnerable to degeneration by this mechanism and promote α-synuclein uptake upon viral neuroinflammation.

Increased α-synuclein levels restricts infection of the brain by RNA viruses following West Nile virus and SARS-CoV-1 infection (59). Due to the similarity of SARS-CoV-2 to these other viruses, it is possible that a similar up-regulation of α-synuclein might occur with SARS-CoV-2 infection leading to widespread neurodegeneration (60).

α-Synuclein is recognized by a number of toll-like receptors (TLRs) including TLR1-4,−7 and−8. TLRs detect pathogen- or damage-associated molecular patterns (PAMPs and DAMPs) that accrue due to cell damage and can trigger a chronic inflammatory process (61, 62). α-Synuclein aggregation also leads to microglial activation, a hallmark of neurodegenerative diseases (63), that further promotes neuronal damage and apoptosis by releasing proinflammatory cytokines and cytotoxic reactive oxygen intermediates (64).

Hyposmia and gastrointestinal manifestations, which are common in SARS-CoV-2 infection, are non-motor symptoms of PD during the prodromal phase. It is in this phase of the disease that α-synuclein deposition within the anterior olfactory nucleus and neurodegeneration begins (65–67).

Furthermore, the nucleocapsid protein of SARS-CoV-2 has the potential to physically interact with the mTOR translational repressor, La-Ribonucleoprotein 1 (LARP1) (68). LARP1 plays an important role in the autophagy, glucose metabolism and mitochondrial functions in the neurons of the brain (69).

Brain hypoxia is another common feature among COVID-19 patients with respiratory distress. Hypoxia induces oxidative damage to neural cells and causes widespread neurodegeneration (70). Based on clinical data obtained from two cases reported by Jaunmuktane and colleagues, severe COVID-19 patients may be at a greater risk of microvascular injury and ischemic lesions due to presence of hypoxia (71).

Acute hypoxemia may promote a hypoxic ischemic encephalopathy (HIE) and induce demyelination and production of white matter microhemorrhages. Furthermore, prolonged hypoxia may lead to BBB destruction and leaky capillaries, which promote cerebral microhemorrhages. The defective function and subsequent death of oligodendroglial cells is another consequence of a hypoxic microenvironment within the brain which, in turn, results in demyelination of white matter (72).

Interestingly Hypoxia can impair metabolic pathways in the brain as well and activate immune cells that are usually maintained in a resting state during normoxia. The hypoxia microenvironment in the brain actives hypoxia inducible factor 1 (HIF-1α) which is a key regulator of oxygen homeostasis and triggers adoptive responses under hypoxic conditions (73). HIF-1α down regulates the expression of ACE2 and transmembrane protease serine 2 (TMPRSS2) and so can exert a protective effect by blocking of the cell entry gate of SARS-CoV-2 (74, 75).

However, the upregulation of HIF-1a may act as a double-edged sword during SARS-CoV-2 infectivity. HIF-1α mediates cytokine storm and mass production of proinflammatory cytokines including IL-6 at the inflammatory site and increases the transcription of M1 macrophage-associated genes such as TNF-α, IL-1β, inducible nitric oxide synthase (INOS) at the site of inflammation. In addition, HIF-1α modulates the activation and life span of many immune cells including neutrophils and dendritic cells to promote M1 polarization (76).

The cytokine storm triggers neuronal damage, epithelial and endothelial cell apoptosis, vascular leakage and BBB disruption (77). In rare occasions the cytokine storm may lead to BBB disruption without direct viral invasion (78). Moreover, disruption of the peripheral BBB increases α-synuclein uptake from the circulation into the brain (Figure 1).

Immunity cell activation and the cytokine storm are proposed as key mechanisms driving the pathogenesis and progression of neurodegenerative diseases. For example, in Alzheimer's disease (AD), increased pro-inflammatory cytokines such as IL-1 and IL-6 suppress the phagocytic activity of microglial cells and cause the accumulation and deposition of beta-amyloid A (79). In addition, increased levels of inflammatory mediators including TNF-α are found in the CSF and in brain tissue of patients with PD as well as in the non-human primate models of the disease (80). A similar pro-inflammatory pattern with increased IL-1, IL-6 and TNF-α is seen in the CSF of MS patients and was associated with the severity of damage at the time of diagnosis (81). Interestingly, men with higher plasma levels of IL-6 have an increased risk of developing PD (67, 82). IL-6 is highly overexpressed in severe COVID-19 patients that may suggest a plausible therapeutic target to prevent the neurological consequences and brain damage seen in these patients.

### Control of Inflammation and the Importance of IL-10 in the CNS

Although limiting viral replication leads to a rapid and effective immune response it needs to be tightly regulated to avoid tissue damage. Given the highly specialized functions and limited regenerative capacity of neurons, tight regulation of the CNS immune response is critical (3). The anti-inflammatory cytokine IL-10 limits the expansion of tissue lesions during chronic viral infection of the CNS (83). Indeed, the pathologic process of virus-induced demyelination is alleviated by exogenous IL-10 early after disease onset and highlights its potential for the treatment of viral encephalitis and COVID-19 (84).

CD8 T cells are the major source of IL-10 production in the airways following infection of murine models with influenza A virus (SV5) and respiratory syncytial virus (85, 86). IL-10 is produced transiently by CD8 T cells in the brain of coronavirus-infected mice at the peak of infection. The expression of IL-10 in CD8 T cells; requires a strong antigenic stimulation and signaling through the MAPK pathway. Thus, the production of IL-10 by highly activated CD8 T cells functions to suppress the localized imune response when the viral antigen concentration and inflammatory responses are increased which reduces immunopathological damage (87).

T helper cells (CD4+ Tcells) can produce IL-10 following antigen stimulation and high T cell receptor (TCR) ligation (87). Development of IL-10-producing Th1 cells is dependent upon IL-12 stimulation via STAT4 and ERK-dependent pathways. Repeated TCR triggering leads to maximal levels of IL-10 production by Th1 cells (88). Futhermore, during high dose bacterial infection and during a cytokine storm, macrophages can produce high levels of IL-10 and miR-155 which orchestrate an inhibitory process protecting the host from immune cell-mediated damage (89). In the patients susceptible to a cytokine storm, macrophages and immune cells have significantly lower IL-10 and miR-155 expression following bacterial challenge (89).

Systemic production of IL-10 following the onset of a cytokine storm may result in “immunoparalysis” that is associated with decreased number and reduced function of neutrophils and monocytes (90) in association with pyroptotic death of these cells (77, 91). In addition, the reduced peripheral blood lymphocyte count, that mainly reflects a loss of CD4+ and CD8+ T cells in COVID-19 patients, is highly associated with the severity of disease. The mechanism underlying this process is unclear but it may be due to the ability of SARS-CoV-2 to infect T cells directly.

However, patients that survive the initial cytokine storm may be unable to recover from immunoparalysis. COVID-19 patients with low monocyte surface expression of HLA-DR have a high mortality rate a few days after the onset of sepsis and the cytokine storm. Approaches to reverse immunosuppression should be considered in these patients (92).

### Autoimmunity as a Link of COVID-19 and Neurodegenerative Disease

Viruses can break down the body's self-tolerance and trigger autoimmune reactions in predisposed individuals by multiple mechanisms including structural mimicking or bystander activation (93). Bystander activation is a common mechanism in neurological disorders. The persistent and hyperactive immune response to a viral infection may sequester and expose auto-antigens to the immune system resulting in a localized pro-inflammatory environment (93, 94). Indeed, SARS-CoV-2 may trigger the production of autoantibodies and develop autoinflammatory reactions in genetic predisposed individuals (95). For example, antiphospholipid (APL) antibodies (96), anti-52 kDa and anti-60 kDa anti–Sjögren's-syndrome-related antigen A autoantibodies (Ro-SSA) (97), antinuclear antibodies (ANA) (98) and anti-citrullinated protein antibodies (ACPA) (99, 100) have all been detected in the serum of COVID-19 patients and which may induce future autoimmune disorders.

In addition to the production of auto-antibodies, pre-existing antibodies also may complicate the pathologic condition. Autoimmune reactions can trigger the neurodegeneration process through autoantibodies mediated mechanisms (101). Immunoglobulins (IGs) or immune complexes can be neurotoxic by inducing proinflammatory responses in the brain parenchyma through microglial activation leading to neuronal damage (101, 102). For example IgG in lupus serum induces M1 polarization of brain microglia and inflammatory responses (103). Furthermore, patients with autoimmune rheumatic diseases have a significantly higher risk of neurodegenerative diseases (104) and autoantibodies that target brain specific proteins have been found in the CSF of patients with AD (101, 105).

An autoimmune background is also suggested for PD. In patients with PD, the presence of serum autoantibodies against catecholamine-based melanins have been identified and are responsible for the neuronal loss by autoimmune mechanisms (106). Furthermore, in MS the increased migration of auto-reactive lymphocytes across the BBB results in persistent neurodegeneration (107). Overall, the current evidence suggests that neuronal damage in COVID-19 patients might be secondary to the immune response due to the presence of autoantibodies rather than to a direct cytopathic effect of the virus (99).

Some cases of Guillain-Barrè syndrome have been reported following SARS-CoV-2 infection (108, 109). Guillain-Barrè syndrome is an immune-mediated pathogenicity and mostly develops after a respiratory or gastrointestinal infection (110). Formation of cross-reacting anti-ganglioside antibodies is the major mechanism driving the demyelinating polyneuropathy or a motor axonal neuropathy (110, 111). Influenza A virus also triggers autoimmune CNS damage with the lung acting as an inflammatory niche in which auto-aggressive T cells gain the capacity to enter CNS (107). Thus, the persistence of the virus or of viral proteins may continue to stimulate the immune system and perpetuate chronic demyelination (112). Together, these findings suggest a potential link between neurological defects and autoimmune reactions upon SARS-CoV-2 infection. Hence, induced autoimmune reactions or the persistence of autoantibodies may exacerbate pathological states within the CNS.

### Preexisting Medical Condition in Neurodegenerative Diseases With the Severity of COVID-19

Current evidence suggests that pre-existing CNS comorbidities increase the severity and neurological damage of COVID-19. The association between neurological comorbidity and COVID-19 severity varies according to the presence of neurological disorders and patients with cerebrovascular and cognitive impairments have a higher severity of infection and a greater need for ICU admission (113, 114). Patients with MS exhibit an increased severity of disease and the infection contributes to disease relapses and/or the worsening of neurological manifestations (115, 116).

Preexisting autoimmune diseases and the presence of autoantibodies could also complicate the inflammatory condition and promote brain injury during SARS-CoV-2 infection (96). The impaired BBB integrity in AD and MS patients may allow autoantibodies to more readily access the brain and trigger neuro-inflammation (101, 117). Pre-existing cardiovascular diseases also increase the neurological injuries induced by SARS-CoV-2 (118). ACE2 usage by the virus and receptor shedding reduced cell surface expression of ACE2 and endothelial dysfunction. Dysregulation of ACE2 enzymatic function may also lead to dysregulation of the brain renin-angiotensin system (B-RAS) whilst overexpression of Ang II and dysregulated blood pressure can impact on brain endothelial cell dysfunction (119). High levels of Ang II can also trigger an increase in pro-inflammatory cytokine levels and promote a cytokine storm within the brain (120). This suggests that the presence of cardiovascular comorbidities exacerbates these conditions and may account for the severe neurological injuries induced by SARS-CoV-2 infection.

Age also plays a determinant role in neurological injury since older individuals have reduced expression of ACE2 and higher levels of angiotensin II. SARS-CoV-2 infection may induce a severe inflammatory environment with increased secretion of proinflammatory cytokines which can actively transfer to the CNS and activate glial neuroinflammatory responses and trigger neuronal damage (121, 122). Recent studies highlight the role of malnutrition and vitamin deficiency on the severity of disease. A low level of vitamin D is associated with COVID-19 severity and immune dysregulation that may impact upon neurological damage (123). Finally, the gut-brain axis may also contribute to the neurological comorbidity of COVID-19 since gut dysbiosis influences the formation of the cytokine storm within the brain (124). This suggests that improving the gut microbiota using probiotics may provide a novel opportunity to control neural damage induced by SARS-CoV-2.

## Conclusion

The COVID-19 pandemic is associated with a wide range of pathophysiologies. Although SARS-CoV-2 is known primarily as a respiratory virus, a wide range of neurological symptoms have been associated with this virus. Alongside a neurotropic behavior and neuroinvasive properties, the virus may trigger neurological defects by triggering a cytokine storm.

Pre-existing CNS comorbidities and autoimmune diseases place the patients at a higher risk for developing neurological symptoms upon infection. Although many biological parameters including age, the presence of cardiovascular diseases as well as malnutrition and vitamin deficiency are associated with COVID-19; a causal link has not yet been defined.

Current evidence strongly suggests that patients surviving COVID-19 are at high risk for the subsequent development of neurological disease. There are concerns that even when the pandemic is over, SARS-CoV-2 might persist within the CNS and cause chronic and latent infection in a large proportion of the population including those who suffered only mild respiratory symptoms. Further studies are needed to investigate potential correlations between acute or mild COVID-19 infections and long-term neurological effects in the population.

## Author Contributions

SA wrote first draft. EM revised first draft. MV, JG, and IA revised final version and impact with new comments for final version. All authors contributed to the article and approved the submitted version.

## Conflict of Interest

The authors declare that the research was conducted in the absence of any commercial or financial relationships that could be construed as a potential conflict of interest.

## Publisher's Note

All claims expressed in this article are solely those of the authors and do not necessarily represent those of their affiliated organizations, or those of the publisher, the editors and the reviewers. Any product that may be evaluated in this article, or claim that may be made by its manufacturer, is not guaranteed or endorsed by the publisher.
